# Enhancing collision prediction in older adults via perceptual training in virtual reality emphasizing object expansion

**DOI:** 10.3389/fspor.2025.1652911

**Published:** 2025-11-04

**Authors:** Kazuyuki Sato, Takahiro Higuchi

**Affiliations:** ^1^ Department for the Psychology of Human Movement and Sport, Friedrich Schiller University Jena, Jena, Germany; ^2^ Department of Health Promotion Science, Tokyo Metropolitan University, Tokyo, Japan

**Keywords:** collision prediction, perceptual training, virtual reality, object expansion, aging, interception, road-crossing

## Abstract

**Introduction:**

The ability to predict collisions with moving objects declines with age, partly due to reduced sensitivity to object expansion cues. This study examined whether perceptual training specifically targeting object expansion improves collision prediction more effectively than repeated practice on an identical collision prediction task. Additionally, the study verified whether such training could be employed to improve prediction accuracy in a more realistic context, using a virtual road-crossing scenario.

**Methods:**

Twenty older adults (71.35 ± 6.04 years; 11 females) participated. All tasks were constructed in virtual reality (VR) from a first-person perspective. Pre- and post-evaluation sessions comprised three tasks: a) an interception task assessing collision prediction ability, b) a target-approach detection task assessing the sensitivity of object expansion, and c) a road-crossing task. Participants were randomly assigned to one of two training groups: (a) a time-to-contact (TTC) estimation group (TE-group) or (b) an interception task group (IC-group). For the TE-group, participants repeatedly performed a TTC estimation task within a VR environment setting to isolate object expansion cues. This was achieved by restricting other visual cues and limiting the target's motion to a head-on collision approach. In the IC-group, participants repeatedly performed the same interception task used in the evaluation session.

**Results and Discussion:**

The TE-group showed significant improvement in collision prediction compared to the IC-group, indicating that training focused on the perception of object expansion was more effective than simple repetition of its evaluation task. However, neither sensitivity to object expansion nor the accuracy of road-crossing decisions improved significantly, suggesting that other factors may have contributed to the observed improvement.

## Introduction

The ability to predict collisions is crucial for navigating complex environments while promptly avoiding collisions with moving entities such as pedestrians and vehicles. Previous studies have demonstrated that this ability declines with age, making it more difficult to accurately assess the collision risk with a moving object (1, 2). Indeed, it has been shown that driving (3) and pedestrian-related accidents (4) are more prevalent among older adults. Therefore, improving collision prediction ability may contribute to the prevention of such accidents.

Two key optical variables have been identified as being important in the context of collision prediction: “object expansion” (1, 5–10) and “bearing angle” (6, 11–16). Object expansion refers to the rate of change in an object's size on the retina, which is needed to estimate the time-to-contact (TTC) with an approaching object. For example, the faster the rate of object expansion changes, the shorter the TTC (17). The bearing angle is the horizontal angle between a moving observer and a moving object, and it is used to judge the trajectory of the object. If the observer perceives that the bearing angle remains constant while they are approaching each other, the observer can determine whether their trajectory is on a collision course with a moving object. Thus, the key point for successful collision prediction lies in accurately perceiving these two types of visual cues.

However, sensitivity to these two types of visual cues declines with age, highlighting the need for strategies to compensate for this decline. Andersen and Enriquez (1) proposed the “expansion sensitivity hypothesis,” which suggests that a reduction in the sensitivity to object expansion makes collision prediction more difficult. Their experiments showed that even when expansion was easy to perceive, older adults detected collisions less accurately than younger adults (1). François et al. (18) used a virtual reality (VR) interception task and found that older adults had difficulty maintaining a constant bearing angle while intercepting a moving object, resulting in more erratic and non-linear movement patterns (18). Their findings suggested that the sensitivity to these two types of visual cues is susceptible to aging.

Previous work from our group investigated which visual cues, i.e., object expansion or bearing angle, should be prioritized to improve collision prediction in older adults (19). Based on the affordance-based model (20, 21), which emphasizes the need to accurately perceive both visual cues for successful collision prediction, an interception task used by Steinmetz et al. (22) was implemented in a head-mounted display (HMD)-based VR environment (22). An original part of the study was to leverage the controllability of VR to apply a visual perturbation to each of these visual cues. It was expected that the perturbation would impair performance only in participants who rely on the optical variable to perform a task effectively. A comparison between older and younger adults revealed that performance declined in older adults when a perturbation was applied to the bearing angle, whereas performance remained unaffected when the object expansion was perturbed. This indicates that improving perceptual sensitivity to object expansion is essential for collision prediction in older adults.

The present study investigated whether perceptual training focused on perceiving object expansion—delivered in immersive, first-person VR without a visible self-avatar—is more effective than training that replicates the evaluation task (i.e., the interception task) in enhancing collision prediction ability in older adults. As mentioned above, the age-dependent decline in sensitivity to object expansion was identified as a primary factor underlying the deterioration of collision prediction in older adults. Similarly, previous research has shown that the ability to detect lateral motion remains relatively unaffected by age (23), supporting the rationale for focusing on object expansion in training. To focus on the perception of object expansion, the present study referred to the experimental design employed in a previous study (10), which eliminated the surrounding visual cues and presented only a target approaching from the front. This design effectively removed bearing angle information, ensuring that object expansion served as the sole visual cue for detecting potential collisions. Such visual manipulation is also suitable from a perceptual training perspective, aligning with evidence that repeated practice targeting a specific visual cue enhances perceptual sensitivity (24–26). Based on this rationale, the present study hypothesized that perceptual training specifically targeting object expansion would be more effective than repeating the interception task in improving collision prediction in older adults.

Furthermore, to evaluate the transfer of perceptual training to an everyday task, we assessed the accuracy of road-crossing decisions. Previous studies have shown that older adults tend to make more errors in road-crossing decisions (27–36), potentially reflecting reduced sensitivity to optical expansion (37). Therefore, the perceptual training that targets sensitivity to expansion cues may be of practical relevance. In the present study, a highly realistic, first-person VR road-crossing simulation allowed participants to experience traffic scenarios that closely approximate real-world conditions in a safe, controlled, and repeatable manner. Critically, stereoscopic, first-person 3D VR has been reported to enhance realism and presence and to reduce extraneous cognitive load (38, 39). It also supports the formation of accurate spatial representations during navigation (40) and is associated with faster, more precise task execution (41). These advantages enabled us to assess performance in a context that more closely resembles real-life behavior. If perceptual training is shown to transfer to ecological performance, it may represent a viable intervention for reducing collision risk in older adults during daily activities.

## Methods

### Participants

Twenty older adults (71.35 ± 6.04 years, 11 females) were recruited for this study. All participants provided written informed consent and received a bookstore gift card for their participation. Participants were excluded if they had a Mini–Mental State Examination (MMSE) score ≤23 (42) or if they self-reported visual disorders. Because participants were permitted to wear corrective lenses (e.g., eyeglasses or contact lenses) during testing, visual acuity was not an exclusion criterion. Although no detailed assessment for vertigo or vestibular dysfunction was conducted, no participants self-reported vertigo symptoms. Participants were monitored throughout the experiment for signs of vertigo and cybersickness (e.g., nausea, dizziness), and no symptoms were reported during or after testing. As use was restricted to the left-hand controller, participant handedness was not assessed. The Ethics Committee of Tokyo Metropolitan University approved the study protocol (Approval No. H5-13).

### Apparatus

The virtual environment employed in this study was generated using the Unity cross-platform game engine (Unity Technologies, San Francisco, US) on a G-Tune E5 laptop computer (Mouse Computer Co., Ltd., Chuo-ku, Tokyo, Japan) with two NVIDIA® GeForce RTX™ 3,060 graphics cards with Max-Q design (NVIDIA, Santa Clara, CA, USA), a 2.3 GHz Core i7-12700H processor (Intel, Santa Clara, CA, USA), and 32 GB of RAM running Windows 11 (Microsoft, Redmond, WA, USA). Participants wore a head-mounted Oculus Quest 2 display (Meta Platforms, Inc., Menlo Park, CA, USA), which has an LCD resolution of 1,832 × 1,920, a refresh rate of 72–120 Hz, a 110-degree viewing angle, and controllers. The height of the viewpoint in the VR environment was 1.2 m above floor level. Only the left-handed controller was used.

## Protocol and tasks

### Procedure

**Figure 1:**
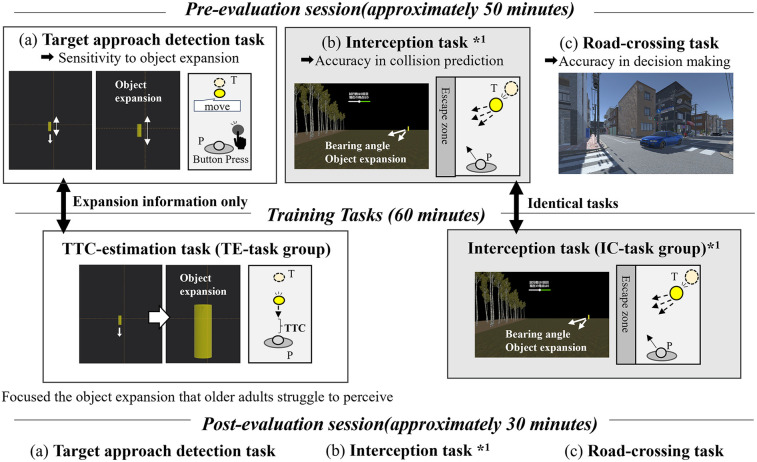
Experimental protocol. P: participant, T: target, TTC: time-to-contact. The evaluation sessions include **(a)** an interception task assessing collision prediction, **(b)** a target-approach detection task assessing sensitivity to object expansion, and **(c)** a VR road-crossing task. The same interception task was used for both the pre- and post-evaluation (*1). The pre- and post-evaluation sessions comprised identical tasks. In the target-approach detection task and the TTC estimation task, only a target approaching directly from the front was presented. In contrast, the interception task included not only the approaching target but also the surrounding environmental visual cues, and the target could move laterally to the left or right.

The experimental protocol is shown in Figure 1. Participants were randomly assigned to one of two training groups: the TTC estimation group (TE-group) and the interception task group (IC-group). Of the 20 participants, 10 were assigned to the IC-group (72.8 ± 5.78 years, 5 females), and 10 were assigned to the TE-group (69.3 ± 3.91 years, 6 females). The experiment consisted of three main sessions: a pre-training evaluation session, a 60 min perceptual training session, and a post-training evaluation session. Each evaluation session comprised three tasks: (a) a target-detection task, (b) an interception task, and (c) a road-crossing task. The same interception task was used for both the evaluation and training sessions in the IC-group. All tasks were conducted in VR using an HMD. Tasks (a) and (b) were performed in an abstract VR environment with handheld-controller input, whereas task (c) was performed in a realistic road-crossing simulation in which participants moved physically within the virtual space. The HMD provided a first-person view, and the scene updated in real time with head movements via head tracking. The entire protocol required approximately 2 h and 30 min to complete. The order of tasks in both the pre- and post-evaluation sessions was counterbalanced across participants to minimize order effects.

## Evaluation tasks

### Evaluation task (a): target-approach detection task

**Figure 2:**
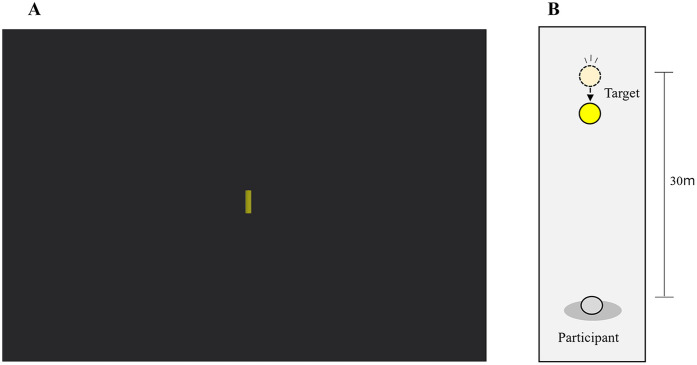
Virtual reality environment used in the target-approach detection task. **(A)** View presented to the participant through the head-mounted display. **(B)** Bird's-eye view of the scene showing the spatial arrangement of the target and participant.

The target-approach detection task was an original task developed to evaluate the sensitivity to object expansion. In this task, participants were instructed to press a button at the moment they perceived the approach of the target, and the reaction time was measured. While seated and wearing the HMD, participants observed only a yellow cylindrical target measuring 1.6 m high and 0.4 m wide against a black background (Figure 2). Because contrast sensitivity declines with age (43–46), high-contrast visual stimuli (yellow cylinders on a black background) were employed in this study to maximize the salience of the expansion cue and minimize floor effects. By presenting only the target and restricting its trajectory to a head-on course, participants needed to rely solely on the visual expansion of the target to detect the approach of the target. Two initial target-participant distances were used: 40 m (far) and 20 m (near distance). Two target speeds were also tested: 100 cm/s (fast) and 50 cm/s (slow speed). Participants pressed a button on a controller with their left hand to indicate detection of the stationary target as it began moving toward them. If the button was pressed while the target remained stationary, a beep sound was played and the trial was considered invalid. The duration of the stationary phase varied randomly between 1 and 15 s across trials. Because the stationary phase before motion onset was widely randomized and premature presses were invalidated, we considered anticipatory response biases to be minimal and therefore did not include dedicated catch (no-motion) trials. The time from the onset of the target approach to the button press was recorded as the detection time. A shorter detection time indicates higher sensitivity to object expansion.

Participants completed 12 trials in total: two distances (near, far) and two speeds (slow, fast), each repeated three times. The average detection time was calculated across all trials. The order of the four conditions (two distances × two speeds) was counterbalanced across participants.

### Evaluation task (b): interception task

**Figure 3:**
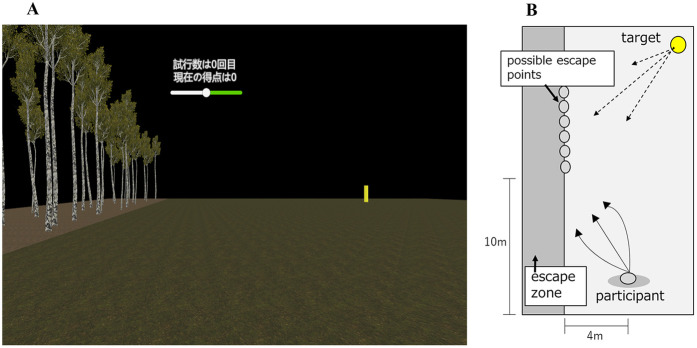
Virtual reality environment used in the interception task. **(A)** View presented to the participant through the head-mounted display. **(B)** Bird's-eye view of the virtual scene showing the positions of the participant, target, and possible escape points.

The interception task was developed based on previous studies (19, 22). Although the target used was the same as in the target-approach detection task, additional visual cues were incorporated into the virtual environment, including a ground surface and a forested area within the escape zone to strengthen optic-flow cues and the perception of self-motion (Figure 3). In this task, participants were required to decide whether to pursue the moving target in an attempt to intercept it or refrain from pursuit if they judged interception to be unfeasible before the target reached the escape zone. A scoring system used in the study of Steinmetz et al. (22) was employed (see Table 1), in which successful interception of the target resulted in point gains, while longer movement distances by the participant incurred point deductions. Participants were instructed to maximize their overall score. Upon a signal from the experimenter, the target began moving toward one of the designated escape points. The speed and direction of the target varied across trials. In approximately half of the trials, the target was programmed to be uncatchable. In these trials, the optimal decision for participants was to refrain from pursuit to avoid score deductions. During the first second after the target motion onset, participants were restricted from moving and required only to observe the target's trajectory (observation phase). At the end of this phase, a beep sound indicated the start of the response phase, during which participants could either attempt to pursue the target or give up. Participants' movement was controlled by using a joystick on the handheld controller, and the decision to give up was executed by pressing a designated button. Each participant completed 30 trials. Before the main trials, participants completed a 50-trial familiarization block to confirm task comprehension and controller proficiency. Because sensorimotor adaptation includes a fast-timescale process that rapidly stabilizes performance (e.g., reductions in movement error) over the first few dozen trials (47), fifty practice trials were deemed sufficient to achieve stable controller use. Further details regarding target speeds, movement directions, escape point positions, practice procedures, and the scoring system are provided in the Appendix 1.

**Table 1:** Trial outcomes and corresponding point values in the interception task.

Measure	Description	Trial outcome	Movement cost	Trial score range
Catch	Participant pursued target until successful interception	Target intercepted (+10 points)	−5 to −8 points	2–5 points gain
Miss	Participant pursued target until it escaped into escape zone	Target escaped (–2 points)	−3 to −8 points	5–10 point loss
Give-up	Participant pursued target then gave up before it escaped	Participant gave up (0 points)	−1 to −5 points	1–5 point loss
No-go	Participant gave up without moving	Participant gave up (0 points)	0 points	0 points

In addition to the scoring system, performance was also quantified based on Signal Detection Theory (48), using hit rate, false alarm rate, sensitivity index (*d*′), and decision criterion (*c*). The hit rate was calculated as the proportion of signal-present trials in which the participant correctly identified the presence of a signal (i.e., number of hits divided by the number of signal-present trials). The false alarm rate was defined as the proportion of signal-absent trials in which the participant incorrectly responded as if a signal were present (i.e., number of false alarms divided by the total number of signal-absent trials). The sensitivity index *d*′ was calculated using the following formula (49):

$$d^{\prime }\;=\;Z\;(\mathrm{Hit}\;\mathrm{rate})-\;Z\;(\mathrm{False}\;\mathrm{alarm}\;\mathrm{rate})$$

A higher *d*′ value indicates greater discriminability in decision-making. For example, frequent misjudgments, such as attempting to pursue an uncatchable target (i.e., a “Go” response on a signal-absent trial) or failing to pursue a catchable target (i.e., a “No-go” response on a signal-present trial), would result in a lower *d*′ value.

The decision criterion (*c*) quantifies a participant's response bias. Whereas *d*′ value reflects the ability to discriminate between signal and noise, *c* indicates the tendency to respond conservatively or liberally to the presence of a signal. The decision criterion was calculated using the following formula:

c=−1/2[Z(Hitrate)+Z(FalseAlarmrate)]

Lower *c* values reflect a more liberal response bias, indicating that the participant is more likely to judge a signal as present based on limited evidence, which increases the likelihood of false alarms. In contrast, higher *c* values indicate a more conservative bias, making the participant less likely to respond to the presence of a signal, increasing the likelihood of misses. A *c* value close to zero indicates a neutral decision criterion with minimal response bias.

### Evaluation task (c): VR road-crossing task

**Figure 4:**
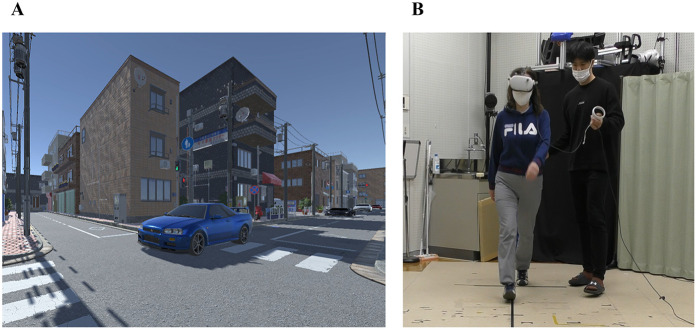
**(A)** Virtual reality scene of a pedestrian crosswalk in a Japanese cityscape, including vehicles. **(B)** A participant performing the task, wearing a head-mounted display, accompanied by an experimenter to ensure safety during actual walking.

A road-crossing task was developed in a VR environment based on the experimental design of Stafford et al. (37), with the primary objective of evaluating the accuracy of participants' road-crossing decisions. The VR environment was designed to realistically replicate a typical Japanese urban street (Figure 4). In this task, participants observed the gaps between five passing vehicles and determined whether they could safely cross the road at their normal walking speed. The size of each gap was determined based on each participant's pre-measured walking speed. Gap sizes were individually adjusted so that one of the gaps was crossable, while the remaining seven gaps were non-crossable gaps. Participants needed to physically cross the virtual road at their normal walking speed while being immersed in a VR environment. Participants were allowed to increase their walking speed during crossing if a collision appeared imminent. If participants judged that the gaps were too narrow to cross safely, they were instructed to wait until the last vehicle had passed before crossing. Each participant performed a total of 15 trials.

To assess decision accuracy, the percentage of trials in which participants correctly identified the crossable gap was calculated. Further details regarding the VR environment setup, vehicle speeds, inter-vehicle gap specifications, walking speed measurement procedures, and familiarization sessions are provided in the Appendix 1.

## Training tasks

### Training task (a): TTC estimation task (TE-group)

The TTC estimation task was developed based on a previous study (50–53). While seated and wearing the HMD, participants were instructed to estimate the time remaining until a target, approaching from the front, would arrive. Participants were required to press a button on the controller at the exact moment they judged that the TTC matched a pre-designated duration (1, 3, or 5 s), with the goal of minimizing estimation error.

**Figure 5:**
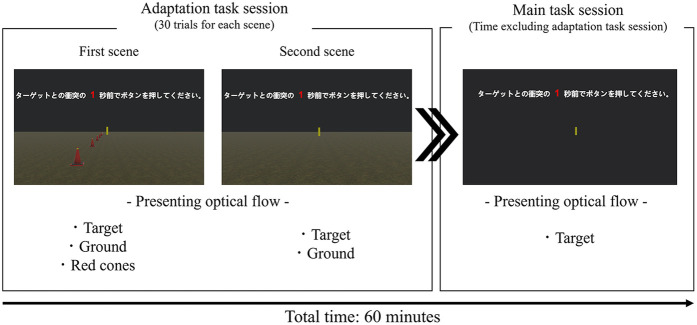
Virtual reality scene used in the TTC-estimation task for the TE-group. The image shows the gradual reduction of visual information (optical flow) from the adaptation task session to the main task session. The total time for the adaptation task session and the main task session was approximately 60 min.

The virtual environment used in this task was identical to that of the target-approach detection task (Figure 5, main task session). To ensure that participants relied solely on object expansion cues, the target approached directly from the front without any horizontal (lateral) movement. The initial distance between the participant and the target varied randomly between 40 and 80 m across trials. The target's approach speed also varied randomly across trials, ranging from 6.8–16.2 m/s (i.e., 24.48–58.32 km/h). The target began moving directly toward the participant following a verbal cue from the experimenter. Immediately after the participant pressed the button, feedback indicating the TTC estimation error was displayed on the screen. Upon confirming the feedback, the participant received a cue from the experimenter to proceed to the next trial. The three TTC conditions (1, 3, and 5 s) were randomized every 10 trials, with the designated TTC duration verbally announced before each block. The current TTC condition was also displayed at the top center of the screen throughout each trial. Participants completed multiple 60-trial blocks within an approximately 60-minute training period, with rest periods provided as needed based on individual fatigue.

Given concerns that detecting object expansion without background cues might be too difficult for older adults, an adaptation task session using two different scenes was performed prior to the main task session. Initiating the TTC estimation task with some additional visual cues was expected to facilitate participants' adaptation to the task (see Figure 5: Adaptation task session). In the first scene, the target was presented against a black background on a green surface. Additionally, red cones were placed every 5 meters to enhance the perception of optical flow. In the second scene, the target remained on the green surface but without the red cones, thereby emphasizing reliance on object expansion cues for TTC estimation. By gradually removing visual cues such as the red cones during the adaptation phase, a smooth transition was provided to the training environment in which the participants ultimately depended solely on object expansion. Participants completed a total of 30 trials, comprising 10 trials for each of the predetermined TTC conditions (1, 3, or 5 s).

### Training task (b): interception task (IC-group)

The interception task used for training in the IC-group was identical to that employed during the evaluation phase. The visual environment, difficulty settings, and task rules were all held constant. Participants were instructed to maximize their total score over 30 trials. The current score was continuously displayed at the top of the screen during each trial. Total scores were calculated at the end of each 30-trial block, and the score was reset at the start of each new block. Participants performed multiple 30-trial blocks within an approximately 60-minute training period, with rest periods provided based on individual fatigue.

### Data analysis

The dependent variables were as follows: target detection time from the target-approach detection task; score and signal detection measures (hit rate, false alarm rate, *d*′ value, and *c*) from the interception task; and decision accuracy from the road-crossing task. For each outcome variable, a two-way repeated measures ANOVA was calculated, with Session (pre, post) as the within-subjects factor and Group (TE-group, IC-group) as the between-subjects factor. In addition to *p*-values, we also reported partial eta squared (partial η^2^). All statistical analyses were conducted using IBM SPSS Statistics for Windows, version 27.0 (IBM Corp., Armonk, NY, USA), with the significance level set at *p* ≤ .05.

## Results

### Target-approach detection task

**Figure 6:**
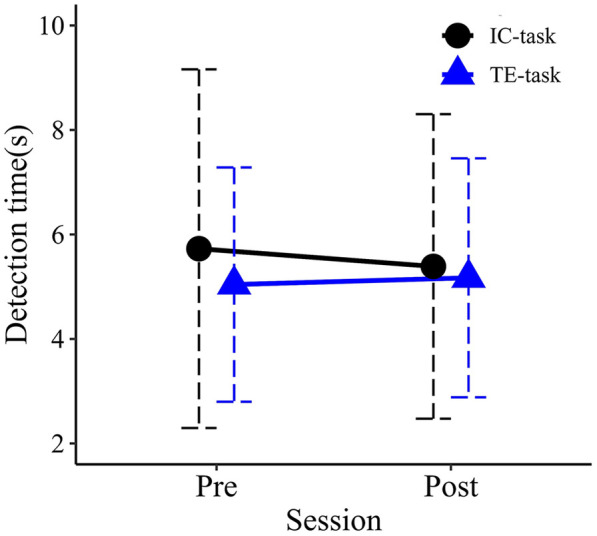
Mean detection times in the pre- and post-training. Error bars represent standard deviations.

Mean detection times for each group in the pre- and post-evaluation sessions are shown in Figure 6. No significant main effects were observed for Session (*F*_1,18_ = .042, *p* = .840, partial η^2^ = .002) or Group (*F*_1,18_ = .162, *p* = .692, partial η^2^ = .009). The interaction between Session and Group was also not significant (*F*_1,18_ = .208, *p* = .654, partial η^2^ = .011).

### Interception task

**Figure 7:**
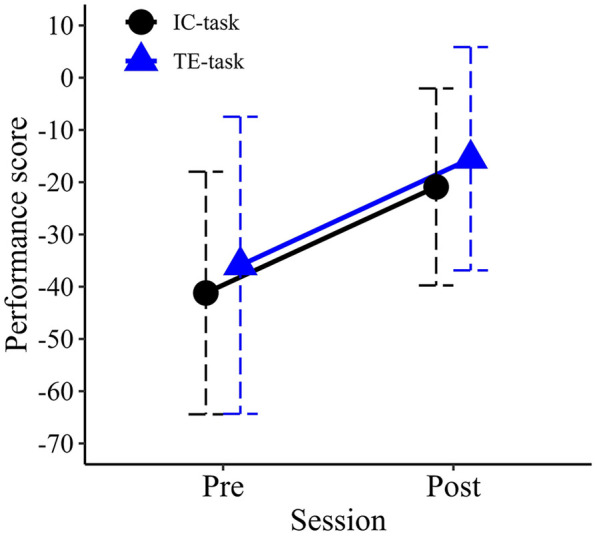
Mean performance scores in the pre- and post-training sessions. Error bars represent standard deviations.

Mean performance scores for each group in the pre- and post-evaluation sessions are shown in Figure 7. An ANOVA revealed a significant main effect for Session (*F*_1,18_ = 21.693, *p* < .001, partial η^2^ = .547), with higher performance scores observed in the post-evaluation session compared to the pre-evaluation session. The main effect of Group was not significant (*F*_1,18_ = .322, *p* = .577, partial η^2^ = .018), nor was the Session × Group interaction (*F*_1,18_ = .000, *p* = .991, partial η^2^ = .000).

**Figure 8:**
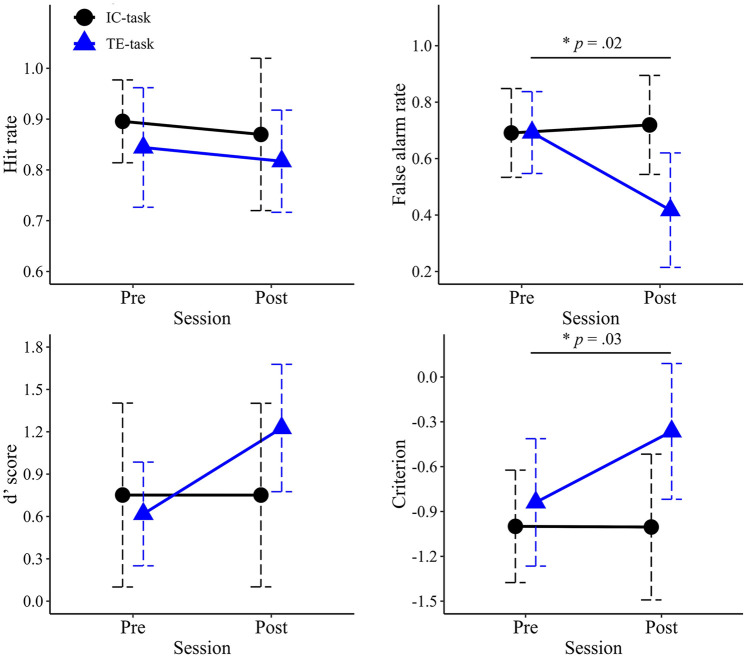
Mean values of signal detection theory measures in the pre- and post-training sessions. The upper left panel shows mean hit rate, the upper right shows mean false alarm rate, the lower left shows the mean d′ value, and the lower right shows the mean criterion. Error bars represent standard deviations.

Mean hit rates for each group in the pre- and post-evaluation sessions are shown in Figure 8. No significant main effects were observed for Session (*F*_1,18_ = .926, *p* = .349, partial η^2^ = .049) or Group (*F*_1,18_ = .000, *p* = .982, partial η^2^ = .000). The interaction between Session and Group was also not significant (*F*_1,18_ = 1.424, *p* = .248, partial η^2^ = .073).

Mean false-alarm rates for each group in the pre- and post-evaluation sessions are shown in Figure 8. A two-way ANOVA revealed a significant main effect of Session (*F*_1,18_ = 7.059, *p* = .016, partial η^2^ = .282), with lower false-alarm rates observed in the post-evaluation session compared to the pre-evaluation session. A significant main effect of Group was also found (*F*_1,18_ = 6.057, *p* = .024, partial η^2^ = .252), with the TE-group exhibiting significantly lower false-alarm rates than the IC-group. The interaction between Session and Group was significant (*F*_1,18_ = 10.688, *p* = .004, partial η^2^ = .373). *post hoc* comparisons indicated that the simple main effect of Group was significant only in the post-evaluation session (*F*_1,18_ = 6.057, *p* = .024, partial η^2^ = .252), where the TE-group demonstrated significantly lower false-alarm rates than in the IC-group.

Mean *d*′ values for each group in the pre- and post-evaluation sessions are shown in Figure 8. No significant main effects were observed for Session (*F*_1,18_ = 2.966, *p* = .102, partial η^2^ = .141) or Group (*F*_1,18_ = 1.036, *p* = .322, partial η^2^ = .054). The interaction between Session and Group was also not significant (*F*_1,18_ = 2.971, *p* = .102, partial η^2^ = .142).

Mean criterion values for each group in the pre- and post-evaluation sessions are shown in Figure 8. A two-way ANOVA revealed a significant main effect of Session (*F*_1,18_ = 5.775, *p* = .027, partial η^2^ = .243), with significantly higher values observed in the post-evaluation session compared to the pre-evaluation session. The main effect of Group was also significant (*F*_1,18_ = 5.565, *p* = .030, partial η^2^ = .236). The interaction between Session and Group was significant (*F*_1,18_ = 5.992, *p* = .025, partial η^2^ = .250). *post hoc* comparisons indicated that the simple main effect of Group was significant only at the post-evaluation session (*F*_1,18_ = 5.565, *p* = .030, partial η^2^ = .236), where the TE-group exhibited significantly higher *c* values than the IC-group.

### Road crossing task

**Figure 9:**
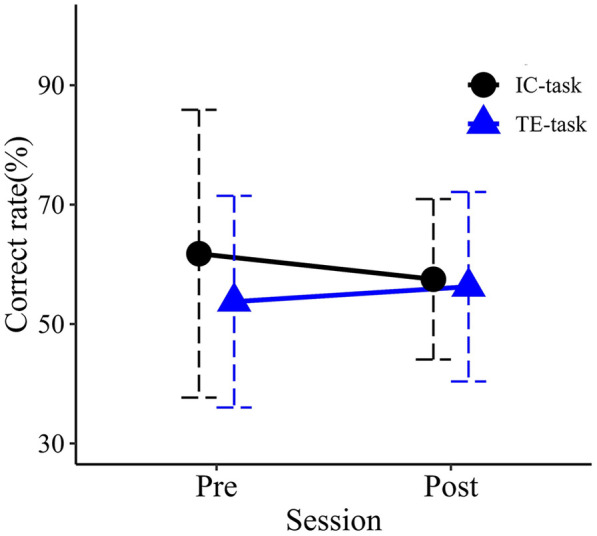
Mean correct rates in the pre- and post-training sessions. Error bars represent standard deviations.

Mean correct rates for each group in the pre- and post-evaluation sessions are shown in Figure 9. No significant main effects were observed for Session (*F*_1,18_ = 0.022, *p* = .883, partial η^2^ = .001) or Group (*F*_1,18_ = 0.699, *p* = .414, partial η^2^ = .037). The interaction between Session and Group was also not significant (*F*_1,18_ = 0.323, *p* = .527, partial η^2^ = .018).

## Discussion

This study examined whether a TTC estimation task designed to enhance sensitivity to object expansion would be more effective in improving collision prediction ability in older adults than repeated practice of the collision prediction task (i.e., the interception task). The results showed that the TE-group exhibited significantly greater improvement in collision prediction performance compared to the IC-group. However, no improvements were observed in sensitivity to object expansion or in the accuracy of road-crossing decisions.

Several previous studies have demonstrated the effectiveness of perceptual training in older adults (54–60). For example, Andersen et al. (56) reported that training with a texture discrimination task improved visual performance in older adults, suggesting that perceptual training may positively influence functional abilities such as driving and mobility. Consistent with these positive findings, the present results indicate that even abilities that decline with age can be enhanced through perceptual training.

**Figure 10:**
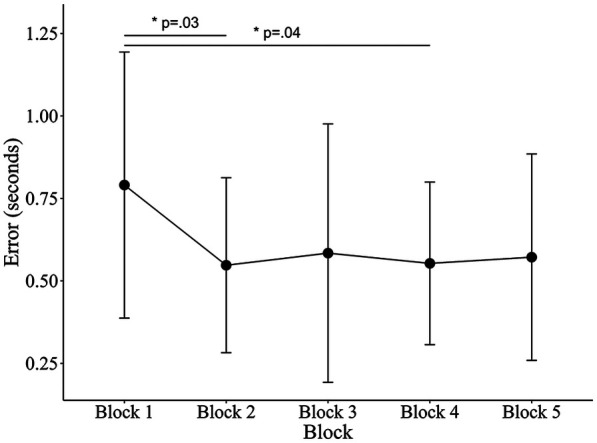
Mean TTC estimation errors across blocks. For each participant, total practice time was divided into five equal segments to account for individual differences in session length. Error bars represent standard deviations. Estimation errors were converted to absolute values. One-way ANOVA showed a significant effect of Block (*F*_4, 45_ = 3.18, *p* = .022, partial η^2^ = .22), indicating that Blocks 2 and 4 were lower than Block 1.

Although collision prediction performance and the accuracy of TTC estimation improved significantly following 60 min of training (see Figure 10), sensitivity to object expansion did not show a significant change. These findings suggest that, contrary to our initial hypothesis, the observed improvements in performance cannot be attributed to increased sensitivity to object expansion. Other factors, such as shifts in decision-making biases or the optimization of cognitive strategies, may have contributed to the improvement observed in collision prediction ability. Previous studies have shown that performance improvements in perceptual tasks can result not only from improvements in sensory processing but also from modifications in decision-making biases (25, 26, 61, 62). For example, Diaz et al. (61) demonstrated that training on a visual categorization task, where participants needed to judge whether noisy images depicted a face or a car, increased neural activity associated more with decision-making than with sensory processing. Similarly, Jones et al. (63) observed a strong correlation between performance improvement in perceptual tasks and reductions in response bias, highlighting the importance of bias control as a key element in perceptual learning (63). In this study, although sensitivity to object expansion did not improve, participants also exhibited reduced judgment bias in the interception task (i.e., improvement in *c*). Taken together, the observed improvement in collision prediction performance may have been rooted in learning mechanisms related to decision-making and response optimization, rather than in perceptual sensitivity improvement.

The absence of improvement in sensitivity to object expansion may be attributable to the limited duration of training. Since a previous study reported that the amount of training is not necessarily critical for learning effects (64) and another demonstrated the effectiveness of a short training session (57), the present study adopted a relatively short training duration (60 min) based on those findings. However, numerous previous studies reporting significant improvements in perceptual performance (55, 58, 65–69) employed training programs of longer duration and distributed over multiple days. It has been shown that spreading training over several days, rather than condensing it into a single session, facilitates memory consolidation during sleep and enhances learning outcomes (70). Based on these findings, future studies may benefit from implementing multi-session training protocols spread over several days while also accounting for the physical limitations of older participants. Such an approach may be more effective for enhancing perceptual sensitivity.

Although collision prediction performance improved in the interception task, no evidence of transfer was observed in the road-crossing task. In general, transfer of training is more likely when the training and real-world tasks are similar (71–74). In the present study, several factors differed among tasks, including attentional demands, situational awareness, psychological factors, and the involvement of actual locomotion. The nature of the visual information presented in the VR environment also differed substantially; for example, a single expanding target was used in the training task vs. multiple vehicles of varying sizes in the road-crossing task. These contextual mismatches may have limited the extent of transfer. Furthermore, the absence of a self-avatar in the VR road-crossing task may also have influenced the results. Presenting a self-avatar enhances presence (75) and improves spatial perception (76), providing a more ecologically valid visual environment. Moreover, because street crossing represents only one component of everyday mobility, it may be beneficial to examine generalization across other ecologically relevant locomotor tasks characterized by differing visual and motor loads.

This study has several limitations. First, we did not assess participant handedness, and all participants used the left-hand controller. Although we provided a practice session, several participants experienced difficulty with precisely controlling the joystick. Therefore, the performance scores in the interception task may have been affected, particularly among right-handed participants. Second, our methodology of using reaction times for evaluating sensitivity to object expansion can be influenced by a variety of factors other than perceptual sensitivity, including motor control, decision bias, and attentional capacity (77–79). As such, even if perceptual sensitivity to object expansion had improved slightly, it may not have been detected through reaction time alone. In addition, including catch (no-motion) trials would have revealed responses to non-events (i.e., false alarms), enabling a cleaner estimate of perceptual sensitivity independent of response bias. Future studies should include catch trials and more direct and sensitive measures, such as discrimination thresholds or neurophysiological assessments like event-related potentials [e.g., visual evoked potentials (VEPs)], to evaluate changes in sensitivity to object expansion more precisely. Third, this study measured the effects of training immediately after the post-assessment session, without examining long-term retention or real-world applicability. Future longitudinal studies are needed to determine how long the training effects persist. Fourth, the relatively small sample limits both generalizability and statistical power. Future studies should recruit larger and more demographically diverse samples. Finally, except for the road-crossing task, the VR tasks were conducted in abstract visual environments, which may limit generalizability.

## Conclusion

The present study demonstrated that training with a TTC estimation task focused on object expansion effectively improved collision prediction performance in older adults. This finding suggests that age-related declines in collision prediction ability can be improved through perceptual training. However, no improvement was observed in sensitivity to object expansion, indicating that the observed enhancement in performance may be attributed to changes in decision-making processes or reductions in judgment bias rather than increased perceptual sensitivity. Furthermore, no improvement was found in road-crossing decisions, a more practical and complex task, suggesting limited transfer of learning to real-world situations. Future research should focus on developing practical training programs that can contribute to performance improvements in real-world situations.

## Data Availability

The raw data supporting the conclusions of this article will be made available by the authors, without undue reservation.

## Appendix 1

### Details of the evaluation tasks

#### A Target detection task

Before the main trials of the target detection task, participants completed two types of practice sessions. First, to familiarize participants with detecting the onset of target motion and pressing the response button, three practice trials were conducted using a clearly detectable target approaching at a high speed (300 cm/s). Second, to discourage premature button responses (i.e., responding before target motion onset), participants practiced withholding their response until they could clearly detect the target's approach. In this session, the target again approached at 300 cm/s. If the button was pressed while the target remained stationary, a beep sound was emitted to indicate an error. This practice was repeated, including trials with a stationary duration of 20 s, until the participant consistently refrained from premature responses.

#### B Interception task

The interception task was identical to that used in our previous study, with few modifications. Due to a change in the controller specifications, movement direction was no longer determined by the orientation of the HMD, as in our previous study. Instead, in the present study, directional control was implemented using the joystick on the handheld controller. To enhance the perception of optical flow during movement, a forested area was added within the escape zone, and the background was set to black to match the visual settings of the other tasks. Aside from these modifications, all other aspects of the task remained unchanged.

As shown in Figure 3, six escape points were positioned within the escape zone at 1.2-meter intervals. The time from the start signal to the moment the target reached the escape zone, referred to as the escape time, was set to one of five values: 2.3, 2.8, 3.3, 3.8, or 4.3 s. The target's approach angle ranged from 50° to 75°, and its speed varied randomly between 2.3 and 10.9 m/s. The target's initial position was randomized within the range defined by the combinations of speed and angle. Participants always started from a fixed position: 10 meters behind and 4 meters to the right of the nearest escape point. Movement direction was determined by the joystick on the left-hand controller, and the “Give Up” response was triggered by pressing the trigger button on the back of the controller. Movement speed was also controlled by joystick tilt, ranging from 0 to 4.5 m/s. In 43.3% of the trials, the target was programmed to be uncatchable, meaning that even with an ideal reaction and trajectory, interception was not possible.

Regarding the scoring system, participants received +10 points for a successful interception, which was defined as reaching the target at a distance of 1.05 meters or less (defined as a “Catch”). If the target reached the escape zone first, then participants lost 2 points (defined as a “Miss”). In cases where participants abandoned pursuit either after initiating movement (defined as “Give-up”) or without initiating movement (defined as “No-go”), the base score was 0. In addition, participants incurred a movement cost, with 0.5 points deducted for every 0.5 meters travelled.

Before the pre-evaluation session, participants completed a practice block to ensure their full understanding of the task and establish proficient control of the left-hand controller (button press and joystick operation). Each participant was first required to successfully intercept the target in at least 10 practice trials, confirming appropriate operation of the controller. In addition, participants were also required to demonstrate an understanding of the No-go concept and verify proper use of the controller's response button by refraining from pursuing clearly uncatchable targets in 10 trials. Only after confirming that participants had adequately understood the task rules did participants complete a 30-trial preliminary block to familiarize themselves with the scoring system. The main evaluation then commenced.

#### C Road-crossing task

In this experiment, the time required for participants to safely cross between vehicles was estimated by calculating a “*minimum crossing time*” based on both the crossing distance and each participant's pre-measured walking speed. Although the total width of the road was 4.3 m, only the first 2.15 m of that space constituted the vehicle travel lane and posed a collision risk. Furthermore, an additional 0.3 m was added to account for the participant's body width, resulting in a final crossing distance of 2.45 m that is used in the calculation. The *minimum crossing time* was determined by dividing this 2.45 m distance by their normal walking speed. A 1.5-second safety margin was then added to this *minimum crossing time*. The sum of the *minimum crossing time* and this margin was then multiplied by vehicle speed, which was randomly set between 40 and 60 km/h. This calculation yielded the final distance defining a “crossable gap.” One of the four gaps was randomly designated as the crossable gap. In contrast, for uncrossable gaps, a randomly selected time of 0.3–0.6 s was subtracted from the participant's previously calculated *minimum crossing time*.

To facilitate walking in the VR environment, participants first practiced crossing a road several times in a vehicle-free setting. Following this familiarization phase, participants completed three road-crossing trials to measure their normal walking speed, which was calculated using the HMD's positional tracking data. Among the three trials, the fastest walking speed was selected and used as the basis for configuring individual gap timings in the main task. Given the potential risks associated with the road-crossing task, such as tripping, falling, or VR-induced discomfort, an experimenter accompanied each participant throughout the task to ensure their safety.
